# Experiences of Health Care and Psychosocial Needs in Parents of Children with Spinal Muscular Atrophy

**DOI:** 10.3390/ijerph20075360

**Published:** 2023-03-31

**Authors:** Laura Inhestern, Maja Brandt, Joenna Driemeyer, Jonas Denecke, Jessika Johannsen, Corinna Bergelt

**Affiliations:** 1 Department of Medical Psychology, University Medical Center Hamburg-Eppendorf, 20246 Hamburg, Germany; 2 Department of Pediatrics, University Medical Center Hamburg-Eppendorf, 20246 Hamburg, Germany; 3 Department of Medical Psychology, University Medicine Greifswald, 17475 Greifswald, Germany

**Keywords:** spinal muscular atrophy, pediatric, children, genetics, treatment, outcome

## Abstract

Spinal muscular atrophy (SMA) is a neurodegenerative disorder that is characterized by progressive weakness, respiratory insufficiency, and dysphagia. Due to symptom burden and disease progress, its care management and impact on daily life can severely burden the families of affected children. The objectives of this study are (1) to explore the health care experiences and (2) to investigate the psychosocial needs of the parents of children with SMA. In total, 29 parents of patients with SMA participated in our study. All children received supportive therapy (e.g., physiotherapy) and most were dependent on medical equipment. Parents perceived the health care positively regarding team quality, communication and access to medical care. An assessment of the impact of the child’s health on the family (e.g., stressors, burden, consequences) is not routinely integrated into care. On average, parents reported low to medium levels of psychosocial needs. Due to the complex health care needs of SMA patients, the health care experiences of parents can provide relevant information on care delivery. To enhance the inclusion of psychosocial and emotional issues, as well as family impact, into routine health care, health care providers should be sensitive towards parental needs for consistency in the health care team and emotional aspects and, if applicable, address them proactively.

## 1. Introduction

Spinal muscular atrophy (SMA) is a neurodegenerative disease that leads to progressive muscular weakness, respiratory insufficiency, bulbar dysfunction, and orthopedic complications, while cognition and social development are normal [1]. Subtypes of SMA are generally classified based on the severity of symptoms and the age of onset. If untreated, severe SMA subtypes lead to death within early infancy [2]. Parents, as the informal caregivers of children with SMA, have shown a high caregiving burden and a decreased quality of life [3,4].

In recent years, treatment options for SMA have increased and the care concept has changed from a palliative care concept to a disease-modifying treatment [5]. In 2017, the antisense oligonucleotide nusinersen was approved for the treatment of 5q-associated SMA in Europe. The medication is applied via lumbar puncture and has to be continued throughout one’s life. In May 2020, gene replacement therapy (onasemnogene abeparvovec) received marketing authorization in Europe, but few SMA patients were treated in clinical trials and/or with pre-marketing before approval. Risdiplam, which is administered orally as a liquid, was approved in Europe in March 2021 as a third treatment option.

Still, families face complex medical decisions; they may place high hopes on new treatment options and may be in need of additional support [6]. In general, caregiving for a chronically ill and/or disabled child can impact parental sleep, work, and future perspectives [7,8]. The treatment of a child with SMA can be psychologically burdening due to the life-limiting character of the disease [9]. Moreover, hospital stays and invasive procedures, e.g., lumbar punctures or home ventilation, can be stressful for patients and their parents [9]. Psychological support, and access to information, resources and technical aid supplies have been identified as the central needs of families with a child with SMA [9,10]. Patients require treatment via different health care disciplines; indeed, health care management can be highly demanding for parents as informal caregivers. In Germany, health care often is fragmented regarding its setting (inpatient and outpatient setting), or its medical and therapeutic specialists. Therefore, efforts have been made to develop and evaluate integrated care models [11]. Still, it is essential to capture the experiences of the patient and caregiver with regard to health care and their psychosocial needs in routine care settings; this is in order to obtain optimal health care and to evaluate current practices [12,13,14]. Although the experiences of parents living with a child with SMA have been the focus of several studies [15], experiences in healthcare from the patient’s or family’s perspective have rarely been assessed in the context of SMA, or have only focused on single aspects, e.g., information or treatment decisions [16,17,18,19].

Therefore, the aims of this study were (1) to investigate the integrated care experiences of parents of children with SMA and (2) to identify the psychosocial needs of affected families. The results might give an insight into the situation of families with a child with SMA and provide a basis for further health care planning.

## 2. Materials and Methods

The presented results are a cross-sectional analysis based on the baseline data of a study with three measurement time points (baseline, 1-year follow-up, 3-year follow-up) in a neuropediatric specialty ward for children with SMA. The study was approved by the local psychosocial ethics committee.

### 2.1. Participants and Procedure

We included pediatric patients with SMA treated in the Department of Pediatrics. Patients and their caregivers were invited to participate in our study if they had an appointment/hospital stay. The recruitment period for baseline data was between April 2020 and January 2022. The inclusion criteria were as follows: children diagnosed with SMA (type I–III), children aged between 0 and 18 years and children treated at the neuropediatric specialty ward. Parents with insufficient language skills, parents presenting a self-assessment that reported a burden too high for study participation, and patients/parents not providing informed consent were excluded. In case of study participation, written informed consent was obtained from caregivers and children >8 years. Parents received a questionnaire including a set of instruments. Children >8 years were invited to provide additional self-report data (not presented within this publication). In case of an inability to provide written answers, children received support to fill out the baseline questionnaire from a member of the study team.

All parents included between April and September 2020 were also invited to participate in an additional interview study at the first measurement point (qualitative data not presented within this publication).

### 2.2. Measures

Sociodemographic characteristics were assessed using the parental report. Additionally, parents provided information regarding disease-related variables (SMA type, time since diagnosis, treatment received). The SMA type was characterized by the time of the first symptoms, the best motor function (not sitting, sitting or walking) and SMN2 copy numbers.

#### 2.2.1. Pediatric Integrated Care Survey (PICS)

Patient experiences of health care were assessed using the Pediatric Integrated Care Survey (PICS). So far, it is the only validated instrument in Germany to assess experiences of integrated care.

Items refer to the overall health care experiences of patients/parents regarding their care network in the previous 12 months. Parents received a short explanation about the definition of “care network”, which includes all members of their individual child’s care network (e.g., therapists, nurses, pediatricians, specialists).

The original version was developed by Ziniel and colleagues, and comprises a core set of 19 experience items with five subscales (access, communication, family impact, care goal creation, team functioning) [20]. In addition, items regarding health care status and usage are included. In the German version of the PICS, psychometric analyses of the core set revealed a three-factor structure comprising thirteen items on the following subscales: (1) team quality and communication (e.g., “How often have the care network members explained things to you in a way that you could understand?” or “How often have you felt that care net-work members thought about the “big picture” when caring for your child, meaning dealing with all of your child’s needs?”); (2) family impact (e.g., “How often have the care network members talked with you about how care decisions for your child will affect your whole family?”); and (3) access to care (e.g., “How often did you have difficulties getting medical or social services (e.g., therapies, integration assistance, etc.), because there were waiting lists or other problems getting appointments?”) [21].

#### 2.2.2. Parental Needs Scale

To assess the psychosocial needs of families affected by a rare pediatric disease, we translated the psychosocial needs scale originally developed by Pelentsov and colleagues [22,23]. The PNS consists of 15 items on a five-point likert scale (1 = no need/extremely satisfied, 5 = high need/not satisfied). Following the analytic strategy of the authors of the original version of the PNS, items were summed up on the subscales according to the originally postulated subscales (understanding the disease, working with health care professionals, emotional issues, financial needs). After that, sum scores of the subscales were transformed to a scale from 0 to 25. Subscale scores were summed up revealing a total score ranging from 0 = no need/extremely satisfied to 100 = high need/not satisfied.

### 2.3. Analyses

Statistical analyses were conducted using the statistical package for the social sciences (SPSS Statistics, Version 27.0, IBM Corp., Armonk, NY, USA). Descriptive statistics were computed for sociodemographic, disease-related data and study outcomes. We used mean and standard deviation for continuous data and frequencies, and percentages for categorical data. Due to the small sample size, differences between SMA subtypes in patient experiences and parental needs were investigated exploratorily using univariate analyses of variances (ANOVAs).

## 3. Results

### 3.1. Sample Characteristics

Of 49 potentially eligible families, the parents of 29 families participated in our study. Reasons for non-participation were insufficient language skills (n = 10), an excessive burden (self-assessment) (n = 8) or other/unknown (n = 2).

Most participating parents were mothers (86%), with a mean age of 39.1 years (SD = 13.8) (Table 1). Children were, on average, 6.2 years old (SD = 5.3). Most children had SMA type 1 or type 2 (Table 1).

### 3.2. Health Care Usage and Status

All children but four had received treatment with nusinersen (Spinraza^®^) within the months prior to the study. Five children had received gene replacement therapy with onasemnogen abeparvovec (Zolgensma^®^). Two of them had previously received nusinersen. All children had received physiotherapy, speech/language therapy or occupational therapy. Almost all children needed medical equipment (e.g., ventilation) (79%). In most cases, pediatricians (97%) and at least one specialist (72%) were involved as healthcare providers in the child’s health care. In 79% of the families, the child attended routine check-ups at the pediatrician. More than half of the children had to visit the emergency department at least once within 12 months prior to the study (Table 2).

With regard to medical or social services, some parents reported having difficulties in getting appointments or that responsibilities were unclear. Almost all parents reported that their children were limited in doing things most children of the same age do (93%), and that their children needed more medical and supportive care than most children of the same age (83%).

### 3.3. Experiences of Care

Parents rated their experiences with health care regarding team quality and communication with M = 30.4 (SD 6.3) on a possible scale ranging from 7 to 42, indicating well-perceived care (Table 3). Parental reports indicate that the assessment of the impact of the child’s health on the family (e.g., stressors, burden, consequences) was not routinely integrated into care (M = 9.9, possible range 4–24 with higher values indicating higher integration). Parents reported few difficulties with regard to access to medical or social services/care (M = 4.8). The exploratory analyses did not reveal any significant differences between SMA subtypes in the subscales.

### 3.4. Parental Needs

On average, parents reported low to medium levels of need across all items of the parental needs scale (Table 4 and Table 5). The lowest levels of need were reported with regard to understanding the disease. Parents reported medium levels of satisfaction regarding the scale related to working with health care professionals. A group comparison between SMA types did not reveal any statistically significant group differences (Table 5).

## 4. Discussion

This study investigates the health care experiences and psychosocial needs of parents with children diagnosed with SMA. All SMA patients but one in our sample received drug treatment, mostly nusinersen, and few patients were treated with gene replacement therapy. The findings reveal a mixed picture of parent-reported experiences of health care. Similar to the findings on the subscale “team quality and communication” of the original authors of the PICS, parents in our sample experienced this aspect of care positively, with M = 30.4 on a scale with a maximum of 42. Generally, parents perceived the communication with professionals involved in the health care of their children as empathetic. Moreover, they considered the health care team to adequately address main aspects of their child’s care (e.g., passing treatment recommendations within the team, being aware of the “big picture” when caring for the child). Particularly, since a new treatment option was approved shortly before recruitment, parents seemed to feel informed and involved. Parents experienced rather easy access to care, only few indicating difficulties in getting appointments or the information they needed. As the average time since diagnosis was 64 months, parents in our sample seemed to be—possibly due to their extensive experience—well informed about the processes involved in health care and supportive care for their child, about central contact persons and about trustworthy sources of information. Particularly, shortly after diagnosis, families may need more support in navigating through the health care system and obtaining access to information about the disease [24].

According to the parents, the impact of the disease and the treatment on the family (e.g., stressors, burden, consequences) was not routinely addressed in health care. The involvement of psychosocial support (psychologist, social worker) was not reported. This finding is similar to other studies, identifying a lack of routine psychological care [10]. The reasons for this might include parents missing routinely implemented support offers within the clinics or long waiting times in outpatient support offers (e.g., psychotherapists). Moreover, health care teams might focus on medical care or their specialty, and typically have limited or missing staff resources for the psychosocial support of the families. At the same time, parents report a medium psychological need regarding emotional issues. Previously, parents of children with SMA have reported emotional burdens, such as being confronted with premature death, uncertainty or fears [25]. Current findings show high levels of caregiver strain [8]. Offering routine, and low-threshold psychological support for affected children and their parents could unburden the families and prevent long-term mental consequences [26]. To deliver comprehensive integrated care, these aspects should be assessed routinely by care providers, as parents may not proactively address these aspects themselves [27,28]. In many cases, patient organizations are an important part of providing peer support and psychosocial support [26,29].

Compared to the findings of the validation study of the parental needs scale from Australia and New Zealand, including a parent sample of children with mixed rare conditions [22], our findings indicate less needs for psychosocial support. While the majority of parents report low to medium levels of need in terms of emotional aspects, several parents still report a high emotional burden (sadness, depressed, frustrated). Moreover, eight families did not participate due to an extensive burden. Therefore, we might underestimate the actual level of need regarding emotional burden. The highest needs were reported in the subscale “working with health care professionals”. This might reflect the parental wish for a consistent health care team or the wish to be treated as an integral part of the health care team. Our study sample reported that the parents had little need in terms of understanding their child’s disease, comparable with a study on the parental needs of parents of hemophilia patients [30]. One reason for this might be that our sample of parents is experienced (average time since diagnosis >60 months before the survey) and they feel confident in explaining the disease of their child. This result underlines findings from qualitative studies, which report that parents become experts in their child’s disease [31].

It has to be considered that, for a long time, SMA was a disorder that meant a palliative care situation for most of the patients and early death in many of them until a treatment became approved in 2017. Currently, three treatment options that considerably improve the disease course are available, but the long-term outcome of the patients is still unknown. Thus, the current study was performed shortly after the approval of an effective therapy with a considerable effect on patients’ and parents’ confidence in terms of the positive course of the disease. The current study represents a special situation shortly after the implementation of a therapy for a former mostly palliative disorder. It is possible that the need for psychosocial support may be underestimated due to an optimistic view of these therapeutic approaches [32]. However, care coordination needs or support in participation and daily life may continue to be relevant.

Since life expectancy and the severity of the disease significantly differ between the SMA subtypes and the age at which children receive treatment, future studies should include different SMA types and larger sample sizes, to systematically analyze group differences.

### Limitations

This study has several limitations. First, we only included a small sample size with a cohort from one specialized center. Hence, the results should be interpreted with caution, since care might be organized and available differently in other clinics or countries, e.g., with regard to resources and staff for routine psychological support. Moreover, the potential differences between SMA subtypes might not have been identified as statistically significant due to the small sample size. Secondly, the majority of participating parents were female. Future studies should include both parents, if applicable, in order to allow a comprehensive insight into the parental situation. Moreover, there might be a selection bias in our sample since we excluded patients/families with insufficient language skills and a too extensive burden to participate.

## 5. Conclusions

Due to the complex health care needs of SMA patients, the health care experiences of parents can provide relevant information for health care professionals on current care delivery, and the patient- and family-centeredness of care. Although team communication and access to relevant care was perceived positively, there are improvements to be made with regard to the impact of the disease on the family and emotional aspects. In light of the rapid development of treatments, it is highly likely that the patient and family perspective on health care and in health care research will gain significance, e.g., in decisional situations [6]. Moreover, parental need assessments allow for the identification of parents with high needs as a basis for needs-based support offers. In order to enhance the inclusion of psychosocial and emotional issues, as well as family impact into routine health care, health care providers should be sensitive towards these aspects and address them proactively. The validation of their situations and their emotions by the health care team, can be a first, basic resource for affected parents.

## Figures and Tables

**Table 1:** Sample characteristics, total n = 29.

Variable		
**Parents/Family characteristics**	n	%
Female	25	86.2
Male	4	13.8
	M	SD
Age	39.1	8.3
School education	n	%
≤10 years	12	41.4
11–13 years	17	56.6
Living with a partner	24	82.8
(M, SD)	M	SD
Number of children living in the family	2.2	1.6
**Patient characteristics**	n	%
Female	19	65.5
Male	10	34.5
	M	SD
Age	6.2	5.3
(in months, M, SD, Range)	M	SD, Range
Time since diagnosis (in months)	63.9	57.0, 0–164
SMA type ^A^	n	%
Type 1/intermediate Type 1–2	11	38.6
Type 2/intermediate Type 2–3	12	42.9
Type 3	5	17.9

^A^ one missing value.

**Table 2:** Health care usage and child’s health status in the 12 months prior to the study (total n = 29).

Variable	n	%
Treatment received		
Nusinersen	25	86.2
Onasemnogene Abeparvovec	5	17.2
Supportive care/therapies		
Physiotherapy, speech/language therapy or occupational therapy	29	100
Medical equipment (ventilation, agility or nutrition)	23	79.3
Support for emotional, developmental or behavioral difficulties	10	34.5
Health care providers involved in child’s care		
Pediatrician	28	96.6
Specialist	21	72.4
Home care nursing/service	2	6.9
Psychologist/Counselor	-	-
Social worker	1	3.4
Complementary/alternative medicine	5	17.2
School/Kindergarten assistance	3	10.3
Routine check-ups at the pediatrician	23	79.3
Emergency visits		
Never	14	48.3
1–2	10	34.5
3 or more	5	17.2
Inpatient hospital stays		
Never	1	3.4
1–2	8	27.6
3 or more	20	69.0
Access to medical records for all medical providers		
Yes, definitely	22	75.9
Yes, somewhat	7	24.1
Difficulties to get medical or social service appointments		
Never	8	27.6
Rarely	8	27.6
Sometimes	8	27.6
Usually	3	10.3
Almost always/always	2	6.8
Difficulties in medical or social service due to unclear responsibilities		
Never	8	27.6
Rarely	9	31.0
Sometimes	8	27.6
Usually	3	10.3
Almost always	1	3.4
Child’s health limited or prevented him/her in any way to do things that most children of the same age can do		
Yes, definitely	16	55.2
Yes, somewhat	11	37.9
No	2	6.9
Child used or needed more medical or supportive care than most children of the same age		
Yes, definitely	18	62.1
Yes, somewhat	6	20.7
No	5	17.2
Change in health care needs		
Persistently	1	3.4
Frequently	1	3.4
Infrequently	15	51.7
No change	12	41.4

**Table 3:** Health care experiences in families with children with different SMA types (comparison of PICS subscales), n = 29.

PICS Subscale	Total (n = 29) ^C^	Type 1/Type 1–2 (n = 11)	Type 2/Type 2–3 (n = 12)	Type 3 (n = 5)
	M (SD)	M (SD)	M (SD)	M (SD)
Team quality and communication (Range 7–42) ^A^	30.4 (6.3)	29.7 (6.9)	29.9 (6.4)	33.0 (5.2)
Family impact (Range 4–24) ^A^	9.9 (4.3)	10.9 (4.7)	9.5 (4.2)	8.6 (4.3)
Access to care (2–12) ^B^	4.8 (2.0)	4.4 (2.5)	4.4 (1.5)	6.4 (0.9)

^A^ Higher values indicating better integration/better-perceived care. ^B^ Lower values indicating fewer difficulties. PICS, Pediatric Integrated Care Survey. ^C^ one missing value on SMA type.

**Table 4:** Parental needs in families with children with SMA, total n = 29.

Parental Needs Scale	M	SD
**Understanding the disease ^A^**		
Teaching my child about the disease	1.8	1.3
Explaining my child’s disease to other children	1.3	0.7
Explaining my child’s disease to my parents or relatives	1.2	0.6
Responding when friends, neighbors, or others ask questions about my child	1.4	0.8
**Working with health care professionals ^A^**		
The overall support that you get from health professionals for your child	2.4	1.0
Having a consistent team of health professionals taking overall responsibility for your child’s health	2.4	1.0
Feeling that you are part of a health care team looking after your child	2.4	1.2
How much health professionals know about your child’s disease	2.2	1.0
**Support on emotional issues ^A^**		
Grief, sadness, hopeless, depressed	2.4	1.4
Isolated, lonely, alienated, rejected	1.8	1.1
Angry, annoyed, frustrated	2.4	1.4
**Financial needs ^A^**		
Paying for babysitting or respite care	2.2	1.5
Paying for special equipment or special clothing	2.5	1.4
Paying for medical care or therapy	1.7	1.1
**Need for…**	n	%
Marriage/couple counselling	3	10.3
Psychological counselling	10	34.5
Financial counselling	4	13.8
Genetic counselling/family planning	6	20.7
Social legal advice/social worker	9	31.0

^A^ Scale range: 1 = no need/extremely satisfied to 5 = high need/not satisfied.

**Table 5:** Parental needs in families with children with different SMA types (comparison of PNS subscales), n = 29.

	Total (n = 29) ^D^	Type 1/Type 1–2 (n = 11)	Type 2/Type 2–3 (n = 12)	Type 3 (n = 5)
	M (SD)	M (SD)	M (SD)	M (SD)
PNS Subscales				
Understanding the disease ^A^	5.7 (2.3)	6.2 (3.3)	5.3 (1.8)	5.6 (1.5)
Working with health care professionals ^A^	9.5 (3.8)	8.9 (2.6)	9.6 (4.6)	10.4 (4.6)
Emotional issues ^B^	6.4 (3.3)	7.6 (3.7)	4.7 (1.1)	7.6 (4.2)
Financial needs ^B^	5.8 (3.1)	6.1 (4.2)	5.7 (2.1)	5.4 (3.3)
Total score ^C^	27.3 (7.6)	28.8 (9.1)	24.8 (7.7)	29.0 (3.5)

^A^ Possible score 4–20; ^B^ possible score 3–15; ^C^ possible score 14–70; in all cases, higher scores indicate higher need/less satisfaction. ^D^ one missing value on SMA type.

## Data Availability

The data that support the findings of this study are available from the corresponding author on reasonable request and after consultation of the local data protection manager. The data are not publicly available due to privacy and ethical restrictions.
